# EQ-5D-5L in Schizophrenia: differences between patients and nurses’ reports

**DOI:** 10.1186/s12955-021-01873-y

**Published:** 2021-10-12

**Authors:** Fredrick Dermawan Purba, Yusi Anggriani, Tri Murtini

**Affiliations:** 1 Department of Developmental Psychology, Faculty of Psychology, Universitas Padjadjaran, Jatinangor, Indonesia; 2 Center for Health Technology Assessment, Universitas Padjadjaran, Jatinangor, Indonesia; 3 Center for Psychological Innovation and Research, Faculty of Psychology, Universitas Padjadjaran, Jatinangor, Indonesia; 4 Faculty of Pharmacy, University of Pancasila, Jakarta, Indonesia

**Keywords:** EQ-5D, Self-report, Proxy-report, Schizophrenia, Indonesia

## Abstract

**Purpose:**

To examine the differences between patient-reports and proxy-reports by nurses of EQ-5D-5L responses among patients with schizophrenia.

**Methods:**

This study was conducted in June–September 2019 in Duren Sawit Regional Public Hospital in Jakarta, Indonesia. The self-report data were obtained by interviewing the patients and the proxy-report data were obtained from the psychiatric nurses. The patients’ Positive and Negative Syndrome Scale (PANSS) scores were obtained from their medical records. The data were collected in two time points: (1) when the patients moved from the acute to the quiet rooms (first-test) and (2) when they were discharged from the hospital (second-test). The self and proxy report scores were analysed by the Wilcoxon matched-pairs signed-ranks test and their relationship with the PANSS scores using Spearman's rank correlation coefficient.

**Results:**

There were 206 patients in the final sample. The majority are male (56.8%) with a mean age of 37.5 years (SD = 12.05). Significant differences between the two reports were found in three domains (i.e., self-care, usual activities, and pain/discomfort) in the first-test and two domains (i.e., usual activities and pain/discomfort) in the second-test. Concerning the relationship with the PANSS scores, only three significant correlations were found, all in the proxy-version and in the second-test: mobility (r = 0.139), anxiety/depression (r = 0.2523), and utility scores (r = − 0.176).

**Conclusions:**

The poor-to-fair agreement between patients and nurses reports and the poor correlation with the PANSS scores suggested that it is difficult to decide which report best represents the patients’ health status.

## Introduction

The resources related to health services: e.g., people, technology. knowledge, budget, are scarce, and choices have to be made. Economic evaluation evaluates alternative policies, services, or interventions which are intended to improve health, in order to ensure the optimal use of health resources for the population [1]. Cost-utility analysis (CUA) is the most widely used form of economic evaluation, by evaluating different health policies, services, or interventions in terms of their cost per Quality Adjusted Life Years (QALYs). To obtain a QALY, utilities of a health state were differentiated over a lifetime. This utility measured by generic multi-attribute utility instruments (MAUIs) [1]. The EQ-5D questionnaire, provided by the EuroQol Group, is the most preferred MAUI to be used in CUA in the national guidelines accross the world [2]. The EQ-5D questionnaire consists of five items covering five dimensions: mobility, self-care, usual activities, pain/discomfort, and anxiety/depression [3]. The so-called descriptive system constructed from these dimensions can be converted into utility scores by applying health preference weights elicited from a general population [4]. It has been found to be valid and reliable in various physical conditions, e.g., cancer [5], stroke [6], type 2 diabetes mellitus [7].

Evidence of performance of EQ-5D in mental health conditions, especially schizophrenia, has not been fully confirmed. Several studies investigating the validity and reliability of different questionnaires, including the EQ-5D, in patients with schizophrenia have shown mixed results [8–11]. This might be related to the effect of schizophrenia on the patients’ cognitive, affective, and reality-testing functions which results in the inability to rate their health status measures properly [12]. In this instance, the assessment of health status of the patients has often relied on proxy reports, mostly done by the clinicians or family members whom are considered having some degree of knowledge of the patients’ illness experience [4].

Previous studies compared health status reported by patients themselves and reported by the proxies in various mental diagnosis or conditions, and the results are mixed. Griffiths et al. [13] found poor agreement of EQ-5D dimensions and utility scores reported by people with dementia and their proxies (i.e., resident and staff). Another study in individuals with severe mental disorders using Quality of Life Index-Mental Health questionnaire reported good agreement in clinical aspects, but not in non-clinical/social aspects [14]. Several studies on children and youth found better agreement between the two reports: Dey et al. [15] and Clark et al. [16] reported good intraclass correlation coefficients across all health status scores measured with KIDSCREEN. These mixed results were also found in studies other patient groups [17–19]. Therefore, the comparison of patient and proxy ratings is necessary, especially in patients with schizophrenia where the evidence is still very limited [20].

The application of EQ-5D in severe mental health conditions such as schizophrenia is still scarce, including in Indonesia. Previous studies used WHOQOL-BREF [21], WHODAS [22], or Lehman’s Quality of Life Interview/QOLI [23], and the self-report version was employed in each studies. Considering the importance of providing evidence of the use of EQ-5D in patients with schizophrenia that can be used in future CUAs, including comparison of two versions of self- and proxy-report, this study seeks to investigate the relationship between patient and proxy evaluations of health status in patients with schizophrenia using the EQ-5D-5L questionnaire.

## Methods

This study is part of a cost-utility study on patients with schizophrenia in Jakarta, Indonesia.

### Respondents

Inclusion criteria were the following: (1) diagnosed with schizophrenia, (2) aged 18 years and above, (3) having an adequate command of the Indonesian language (Bahasa Indonesia), (4) in-patient in quiet rooms of Duren Sawit Regional Public Hospital. Exclusion criteria were the following: (1) refusing to participate, (2) having an incomplete medical record, (3) did not complete the treatment phase (drop out) because of any reason, e.g., insisted to go home, referred to other hospitals, etc.

### Procedures

The study was approved by the Health Research Ethics Committee, Rumah Sakit Angkatan Udara dr. Esnawan Antariksa (Sket/207/IV/2019/KEPK). This study was conducted in Duren Sawit Regional Public Hospital in Jakarta, Indonesia from June to September 2019. The data were collected in two time points: (1) when the patients moved from the acute rooms to the quiet rooms (first-test) and (2) when they were discharged from the hospital as outpatients (second-test). The patients were required to complete the EQ-5D-5L questionnaire in their rooms. Afterwards, the researcher asked the psychiatric nurses on duty to complete the EQ-5D-5L questionnaire regarding the patient’s health-related quality of life based on their opinion. The researcher also collected the PANSS score recorded in patients’ medical records in the two time points.

### Instruments

We collected the demographic and clinical data from patients’ medical records, including: gender, age, length of hospitalization, type of insurance, and origin of the patients (i.e. coming from a family or a social institution/*panti sosial*).

Patients' health status was measured by the Bahasa Indonesia version of EQ-5D-5L provided by the EuroQol Group. The translation of EQ-5D-5L was produced under a standardized translation protocol [24]. The EQ-5D-5L is a generic health status instrument which consists of two parts: i) the descriptive system that consists of five dimensions (mobility, self-care, usual activities, pain/discomfort, anxiety/depression), each of which can take one of five responses (no problems, slight problems, moderate problems, severe problems, and unable/extreme problems), and ii) the EQ Visual Analogue Scale (EQ-VAS), which records the respondent’s self-rated health on a 20 cm vertical visual analogue scale with endpoints labelled “the best health you can imagine” and “the worst health you can imagine” [3]. The EQ-5D-5L has been proven to be valid [5] and reliable [25] to be used on the Indonesian population, and the preferred instrument to be used in CUA in Indonesia [26]. For both the measurement’s point of views (i.e. by the patients themselves and from the nurses), we used the self-report version of the EQ-5D-5L, since the proxy-report of the quesionnaire has not been validated in Indonesia.

The symptom severity of patients with schizophrenia was measured using the Positive and Negative Syndrome Scale (PANSS) [27]. It is a 30-item assessment questionnaire which is divided into positive, negative, and general psychopathology subscales. The scale was administered by trained psychiatrists who assess the weight of each item by giving points of 1–7 according to the severity of the symptoms. PANSS has been proven to be valid and reliable to be used on Indonesian patients [28].

### Data analysis

Descriptive statistics were used to describe the patients’ demographic and clinical data: categorical data were analysed using cross-tabulation and means and standard deviations (SD) were calculated for continuous data.

For each patient, we noted when patient and proxy reported different level of severity for one dimension and counted it as inconsistent. For example, a patient reported that he/she has no problem in walking about (level 1 in mobility dimension) but the proxy reported that that patient has slight problems in walking about (level 2). For each patient, the possible number of inconsistent dimensions ranged from 0 (all five dimensions were reported similar by patient and proxy) to 5 (all five dimensions were reported differently by patient and proxy). We then checked the relationship between the number of inconsistent domains with the PANSS scores using Spearman's rank order correlation.

For the health status obtained from EQ-5D-5L, we calculated the percentages of responses for each level of each dimension. This was done for the two versions: i.e., the self-reported and the proxy-versions and the two time points: first-test and second-test. We compared the equality of responses distribution of each dimension between the two versions at each time point using the Wilcoxon matched-pairs signed-ranks test. Agreement on each EQ-5D-5L dimension was evaluated by calculating the percentage of agreement and weighted kappa coefficients between the two versions [29].

The EQ-5D-5L health states were converted into a single utility score using the Indonesian value set [30]. We compared the equality of responses distribution of utility scores between the two versions at each time point using the Wilcoxon matched-pairs signed-ranks test. The intraclass correlation coefficient (ICC) was used to estimate the interrater agreement of utility scores between the two reports. We then checked the relationship between the utility scores of the reports with the PANSS scores using Spearman's rank order correlation. The interpretation of agreement coefficients was based on published criteria as follows: < 0.2 (poor agreement), 0.21–0.40 (fair agreement), 0.41–0.60 (moderate agreement), 0.61–0.80 (substantial agreement), and > 0.80 (perfect agreement) [31]. In addition, we used the Bland–Altman plots for the utility scores to examine visually the agreement between the two reports in first-test and second-test.

All statistical analyses were carried out using the STATA version 13 software.

## Results

Table 1 shows that the majority of the patients were male (56.8%), below 45 years (74.8%), registered in the national health insurance/*BPJS* (100%), and coming from social institutions/*panti sosial* (67.5%). The average (SD) length of hospitalization is about 12 days (3.2).

**Table 1:** Demographic characteristics of patients (N = 206)

Characteristics	Level	n	%
Gender	Male	117	56.8
	Female	89	43.2
Age group (years)	17–25	25	12.1
	26–35	78	37.9
	36–45	51	24.8
	46–55	33	16.0
	56–65	13	6.3
	> 65	6	2.9
Educational level	Not available*	169	82.0
	Elementary school	8	3.9
	Junior high school	14	6.8
	Senior high scool	15	7.3
Insurance	National health insurance	206	100
Origin	Family	67	32.5
	Social institution	139	67.5
		Mean	SD
Length of hospitalization (days)		12.1	3.2

*Data not written on the medical record

As presented in Table 2, we found three dimensions that the response distribution to the self-report version and the proxy-report (as reported by the nurses) were significantly different in the first-test: Self-care, usual activities, and pain/discomfort. In the self-care dimension, more nurses reported that the patients have no problems in washing or dressing themselves (23.30%) compared to the reports made by the patients themselves (19.41%), while the other way around was shown in the usual-activities and pain/discomfort dimensions: more patients reported that they have no problems in doing their usual activities (12.62%) and have no pain or discomfort (58.25%) compared to the reports made by nurses (5.34% and 39.81%, respectively). No significant differences were found for the mobility and anxiety/depression dimensions between the two versions.

**Table 2:** Self and proxy reported health status using the EQ-5D-5L descriptive system and utility scores in the first-test and second-test

Levels/dimensions (%)	Mobility		Self-care		Usual activities		Pain/discomfort		Anxiety/depression		Mean utility score	
	Self	Proxy	Self	Proxy	Self	Proxy	Self	Proxy	Self	Proxy	Self	Proxy
FIRST-TEST (admitted to the quiet rooms)												
No problems	96.60	95.63	19.41*	23.30*	12.62*	5.34*	58.25*	39.81*	0.97	2.91	0.625*	0.596*
Slight problems	1.94	2.91	50.97*	62.14*	73.30*	60.68*	38.35*	42.72*	19.90	18.93		
Moderate problems	0.49	0.49	28.16*	11.65*	12.62*	32.04*	1.94*	16.02*	58.25	54.37		
Severe problems	0.49	0.49	1.46*	2.91*	0.97*	1.94*	1.46*	1.46*	16.99	21.84		
Unable/extreme problems	0.49	0.49	0.00*	0.00*	0.49*	0.00*	0.00*	0.00*	3.88	1.94		
SECOND-TEST (discharged from the hospital)												
No problems	98.54	98.06	56.31	60.68	38.35*	23.30*	92.72*	72.82*	30.58	29.61	0.829*	0.780*
Slight problems	0.00	0.49	42.23	37.86	60.68*	74.27*	6.31*	26.70*	62.62	59.22		
problems	0.49	0.49	1.46	0.97	0.97*	2.43*	0.97*	0.49*	6.80	11.17		
Severe problems	0.97	0.97	0.00	0.49	0.00*	0.00*	0.00*	0.00*	0.00	0.00		
Unable/extreme problems	0.00	0.00	0.00	0.00	0.00*	0.00*	0.00*	0.00*	0.00	0.00		

*Differences between self-report and proxy-report in the respected dimension and utility scores is statistically significant (*P* value < 0.05)

Similar results were found for the second-test: more patients reported that they have no problems in doing their usual activities (38.35%) and have no pain or discomfort (92.72%) compared to the reports made by nurses (23.30% and 72.82%, respectively). No significant differences were found for the mobility, self-care, and anxiety/depression dimensions between the two versions.

Concerning utility scores, we found a significant difference between the self-report and proxy-report in the first- and second-test, where the mean utility scores of proxy-report was always lower than the self-report.

Table 3 shows that majority of patients received different evaluations of health status between themselves and their proxy in individual dimensions level. Only 5.8% and 16.5% of patients in first and second-test, respectively, reported their EQ-5D-5L dimensions severity level scores while their proxies provided the same exact scores. The rest of the patients had at least one dimension of which their report was different with the proxy report. Spearman’s rank order correlation analysis between number of inconsistent dimensions of each patients had and their PANNS scores show significant but weak correlation in the first-test (Spearman's rho = 0.146; *P* value = 0.0358) and insignificant in the second-test (Spearman's rho = 0.125; *P* value = 0.0737).

**Table 3:** Inconsistency of reporting between self and proxy reports in first and second-tests

Inconsistent dimension*	First-test		Second-test	
	Frequency	%	Frequency	%
0	12	5.83	34	16.50
1	55	26.70	67	32.52
2	76	36.89	63	30.58
3	47	22.82	38	18.45
4	16	7.77	4	1.94

*When patient and proxy reported different level of severity for one dimension, it is counted as inconsistent. For each patient, the possible number of inconsistent dimensions ranged from 0 (all five dimensions were reported similar by patient and proxy) to 5 (all five dimensions were reported different by patient and proxy)

**Fig. 1:**
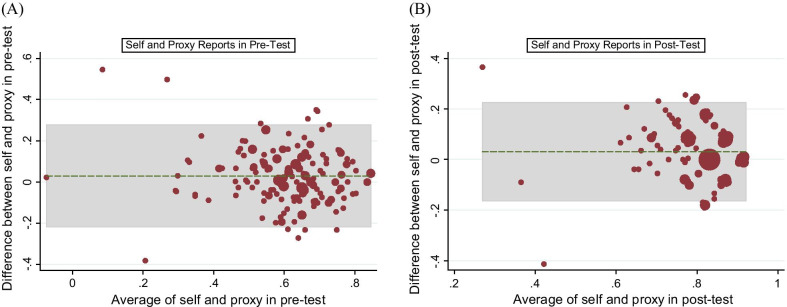
Bland–Altman plot of the utility scores between self and proxy reports: EQ-5D-5L. **a** First-test: 5.8% outside the limit of agreements. **b** Second-test: 7.3%

Table 4 shows results in aggregate level, where the agreement between the two reports shows relatively similar results with that of the equality of distributions. The agreement based on Kappa was perfect for the mobility dimension and poor-to-fair for the other dimensions (self-care, usual activities, pain/discomfort, anxiety/depression) at the first-test and the second-test. For utility scores, the agreement in the first-test was higher than in the second-test but both were still considered as moderate agreement. In addition, inspection of the Bland–Altman plot of the utility scores of the two reports shows that there were 5.8% of first-test and 7.3% of second-test data points where agreement is considered as poor: i.e. lies outside the ± 1.96 SD limits of agreement (see Fig. 1).

**Table 4:** Agreement and Weighted Kappa between self- and proxy-reported health using the EQ-5D-5L descriptive system and utility scores in the first-test and second-test

Dimension	First-test		Second-test	
	Percentage agreement (%)	Weighted Kappa /ICC	Percentage agreement (%)	Weighted Kappa /ICC
Mobility	99.51	0.853	99.64	0.821
Self-Care	89.08	0.406	90.29	0.253
Usual Activities	86.41	0.077	88.83	0.052
Pain/ Discomfort	84.59	0.170	93.93	0.240
Anxiety/ Depression	85.68	0.264	86.77	0.096
Utility score	–	0.603	–	0.482

Table 5 shows the correlation analysis between the self- and proxy-report EQ-5D-5L dimension and utility scores and PANNS scores in the first-test and second-test. We found only three significant correlations with PANNS scores: mobility, anxiety/depression and utility scores of the proxy report in the second-test, with the correlation considered weak (between − 0.176 and 0.252). The other correlations were not statistically significant.

**Table 5:** Correlation between the PANNS scores and self- and proxy-report EQ-5D-5L dimension and utility scores in the first- and second-test

PANNS first-test	Mobility		Self-care		Usual activities		Pain/discomfort		Anxiety/depression		Utility scores	
	Self	Proxy	Self	Proxy	Self	Proxy	Self	Proxy	Self	Proxy	Self	Proxy
FIRST-TEST (admitted to the quiet rooms)												
rho	0.043	0.092	0.029	0.006	0.119	0.063	− 0.01	− 0.065	− 0.062	0.077	− 0.055	− 0.052
*P* value	0.5367	0.1894	0.6755	0.9368	0.0876	0.3690	0.9023	0.3557	0.3765	0.2746	0.4353	0.4567

PANNS second-test	Mobility		Self-care		Usual activities		Pain/discomfort		Anxiety/depression		Utility scores	
	Self	Proxy	Self	Proxy	Self	Proxy	Self	Proxy	Self	Proxy	Self	Proxy
SECOND-TEST (discharged from the hospital)												
rho	0.097	0.139	0.006	0.035	0.045	0.025	− 0.094	− 0.024	− 0.032	0.252	0.001	− 0.176
*P* value	0.1644	0.0470	0.9378	0.6142	0.5184	0.7211	0.1786	0.7340	0.6487	0.0003	0.9955	0.0116

## Discussion

The present study aimed to investigate the relationship between the patient and proxy evaluations of health status in patients with schizophrenia using the EQ-5D-5L questionnaire. We found different results in the dimensions: the patients and the nurses had an almost perfect agreement for the mobility dimension but had poor-to-fair agreement for the other dimensions (i.e. self-care, usual activities, pain/discomfort, anxiety/depression) at the first-test and second-test. In terms of the utility scores, the two reports show moderate agreement in first and second-tests. In term of individual patient analysis, majority had at least one EQ-5D-5L dimensions of which their report (i.e., severity level) was different with the proxy report.

The almost perfect agreement for the mobility dimension (i.e., the ability to walk around) is expected. Similar results are also shown by the previous studies such as in after-stroke patients [32], care home residents [33], patients with dementia [34], and patients before hospital admission [35]. Several explanations offered: (1) ability to walk around is not affected by the schizophrenia, and (2) it is easier for the patients to recognise and report if they have a problem in walking (3) it is easy for the nurses to observe during their daily interaction with the patients.

Self-care, indicated by the patients’ ability to wash or dress themselves, was found to have a fair agreement between patients and nurses, where the first reported more problems than the latter. This dimension is observable, yet a significant difference was reported. It could be argued that patients' disorganized behaviour and/or cognitive disturbance affect their functioning in major areas of their life, such as self-care. However, patients’ act of washing and dressing might not be visible to nurses on a daily basis, hence they considered the patients having fewer problems in this particular dimension.

The usual activities dimension showed the lowest agreement both in the first- (0.0774) and the second-test (0.0524). This is similar to the findings in patients with Dementia [34] and care home residents [33]. A previous study found that the abilities to do housework activities and leisure-related activities were the top two groups of activities patients considered when reporting how their health affects their usual activites dimension of the EQ-5D-5L questionnaire [36]. During their treatment at the hospital, these activities are not feasible to be done. Therefore, it is understandable that 87% and 62% of the patients in the first- and second-test, respectively, reported themselves as having any level of problems in doing these activities. The nurses reported even higher percentages (95% and 77%). These differences may also reflect different perceptions between patients and nurses towards determining which activities covered in the term ‘usual activities’ to focus on.

Poor-to-fair agreement for pain/discomfort and anxiety/depression are also anticipated since the two are not clearly observable. Pain and anxiety are subjective, and it is inherently difficult to judge whether a person is in pain or in anxiety. Concerning pain, the nurses as proxy reported more pain than the patients, and this is similar to other reports from different patient groups [32, 37]. In terms of anxiety/depression, we found that this dimension was reported as the highest percentage of problems from both patients and nurses, similar to what has been found elsewhere [38].

Utility score comparison between the two versions showed a consistent result in two time points: proxy score was significantly lower than the patient score. This finding is in line with the previous studies [17, 33, 39]. Several explanation could be offered: the nurses may overstate the problems patients have based on their own perception of a healthy person or patients included their expectation on their health status and rate their health higher. Further research to explore these discrepancies in detail is warranted.

Despite four out of five dimensions have poor-to-fair agreement, the utility scores resulted from the two reports showed a moderate agreement in the first- and second-tests. One explanation is offered by Kunz who points out that each dimension has different weights in the construction of the utility scores [39]. For example, in the Indonesian EQ-5D-5L value set, the mobility has the highest impact on the utility scores compared to the other dimensions [30]. Therefore, an almost perfect agreement between the patients and the nurses in the mobility dimension could balance the effect of poor agreement in the other four dimensions.

The low inter-rater agreement does not indicate that the nurses as proxy were not able to estimate the patients’ health status, as it is still unclear whether the patients with schizophrenia can be regarded as the gold standard due to their cognitive, affective, and reality-testing functions impairment. This is further supported by the poor correlations between the EQ-5D dimensions scores and utility scores of both versions and both time points with the PANSS scores which could not give a clear evidence which version to choose. However, a systematic review reported mixed results regarding the correlation between EQ-5D and PANSS: several studies found modest and occasionally strong correlations, while several other studies found non-existent or weak correlations [8].

This study comes with several limitations. First, the methodological shortcomings included a relatively small sample size and a cross-sectional design. Second, the selection of inpatients as respondents might limit the generalization of the results of outpatients. Third, the proxy-report were obtained from nurses and not patient’s family members. Further studies might compared three perspectives: patient, family member that taken care of the patient, and nurses/doctors. Fourth, we used the self-report version of the EQ-5D-5L for obtained the health status of the patients from nurses’ point of view. The most suitable questionnaire should be the proxy version, that unforatunately not yet validated in the Indonesian setting. Fifth, we did not collect the visual analogue scale (EQ-VAS) score because the main study was a cost-utility study on patients with schizophrenia, which used only data from the five EQ-5D-5L dimensions. Future study should collect and compare EQ-VAS scores. Sixth, it was difficult to obtain sociodemographic data such as the level of education, job, financial situation, etc. especially from those who were sent to the hospital from the social institutions/*panti sosial* as they usually have no identification card or family contact with them. Seventh, other factors, which were not measured in the present study, may influence the patient–proxy agreement, such as medication side effects, clinical characteristics and insight level. Eighth, EQ-5D-5L is a generic health status instrument, therefore it might not be sensitive to specific aspects relevant to the subjective experiences of patients with schizophrenia. Future studies might plan to include a schizophrenia-specific health status instrument such as Quality of Life Interview (QoLI) or Quality of Life Questionnaire in schizophrenia (S-QoL).

## Conclusion

This study of patients with schizophrenia treated in the quiet rooms of Duren Sawit Regional Public Hospital in Jakarta, Indonesia showed a poor-to-fair agreement between health status of patients measured by EQ-5D-5L questionnaire reported by the patients themselves and the one reported by the psychiatric nurses, except for the mobility dimension. Both versions were also found to be poorly correlated with the PANSS scores. Therefore, it remains inconclusive to choose which report best represents the inpatients with schizophrenia’s health status.

## Data Availability

The datasets used and/or analysed during the current study are available from the corresponding author on reasonable request.

## Declarations

### Competiting interests

FDP is a member of the EuroQol Research Foundation, the copyright holder of the EQ-5D-5L questionnaire.
